# Association of neurotransmitter pathway polygenic risk with specific symptom profiles in psychosis

**DOI:** 10.1101/2023.05.24.23290465

**Published:** 2023-05-28

**Authors:** Tracy L. Warren, Justin D. Tubbs, Tyler A. Lesh, Mylena B. Corona, Sarvenaz Pakzad, Marina Albuquerque, Praveena Singh, Vanessa Zarubin, Sarah Morse, Pak Chung Sham, Cameron S. Carter, Alex S. Nord

**Affiliations:** 1 UC Davis Center for Neuroscience; 2 Department of Psychiatry, The University of Hong Kong; 3 Psychiatric and Neurodevelopmental Genetics Unit, Center for Genomic Medicine, Massachusetts General Hospital; 4 Department of Psychiatry, Harvard Medical School; 5 UC Davis Imaging Research Center; 6 Washington University in St. Louis; 7 Centre for PanorOmic Sciences, The University of Hong Kong; 8 State Key Laboratory of Brain and Cognitive Sciences, The University of Hong Kong

## Abstract

Merging genetic risk, neurological phenotypes, and clinical presentation is a primary goal for psychiatry. Pursuing this goal, we tested association between phenotypes and overall and pathway-specific polygenic risk in patients with early-stage psychosis. Subjects included 206 demographically diverse cases with a psychotic disorder and 115 matched controls with comprehensive psychiatric and neurological phenotyping. DNA was extracted from blood and genotyped. We calculated polygenic scores (PGSs) for schizophrenia (SZ) and bipolar disorder (BP) using Psychiatric Genomics Consortium GWAS summary statistics. To dissect convergent mechanisms of symptoms, we calculated pathway PGSs (pPGSs) for SZ risk affecting each of four major neurotransmitter systems: glutamate, GABA, dopamine, and serotonin. Psychosis subjects had elevated SZ and BP PGS versus controls; cases with SZ or BP diagnoses had stronger SZ or BP risk, respectively. There was no significant association between individual symptom measures and overall PGS. However, neurotransmitter-specific pPGSs were significantly associated with specific symptoms; most notably, increased glutamatergic pPGS was associated with deficits in cognitive control and altered cortical activation during cognitive control task-based fMRI. Finally, unbiased symptom-driven clustering identified three diagnostically mixed case groups with distinct symptom profiles that separated on primary deficits of positive symptoms, negative symptoms, global functioning, and cognitive control. These clusters had specific genetic risk profiles and differential response to treatment, and outperformed diagnosis in predicting glutamate and GABA pPGS. Our findings suggest pathway-based PGS analysis may be a powerful path forward for identifying convergent mechanisms driving psychotic disorders and linking genetic risk with endophenotypes.

## Introduction

Psychosis-spectrum disorders, including schizophrenia (SZ), schizoaffective disorder (SA), and bipolar disorder (BP), affect 0.5-2.3% of people worldwide(1-5). Evidence suggests shared etiology for these disorders, supported by family studies and genome-wide association studies (GWASs) showing high interheritability and shared genomic risk(6-8). Given symptom heterogeneity across the psychosis spectrum, a deeper understanding of the biology underlying specific symptoms may drive psychiatry towards improved patient outcomes using stratified medicine.

One approach toward revealing shared neurobiology is identifying transdiagnostic endophenotypes. Recently, the Bipolar & Schizophrenia Network for Intermediate Phenotypes (B-SNIP) Consortium identified three biotypes of psychotic disorders using neuropsychiatric markers, agnostic to diagnoses(9), that were primarily characterized by differences in cognitive control and sensorimotor reactivity. As knowledge of biology underlying symptom heterogeneity expands, reclassifying psychosis patients using biologically grounded phenotypes may allow for more effective, targeted interventions.

Identification of causal and clinically informative genetic components of psychotic disorders has been aided by large-scale GWASs and estimation of overall genetic risk using polygenic scores (PGSs). While large cohorts were initially required to develop PGSs, once defined, research leveraging PGSs in cohorts with phenotyping that extends beyond diagnosis is revealing how genetic burden is associated with specific symptomatology. For example, recent efforts have found associations between SZ PGS and treatment response(10) as well as neurological and cognitive measures(11,12). Such uses of PGS enable study of genetic burden in small cohorts that have been characterized at a level not feasible at the scale required for GWASs focused on the discovery of new risk loci.

While PGS is a useful metric of genetic risk, it fails to assign burden into relevant biological pathways and elucidate mechanisms underlying symptoms. A novel approach to overcome this is pathway-specific PGS (pPGS), which partitions variants into those contained only in genes of a given function or pathway(13). By assigning burden into pathways, it is possible to test how specific processes contribute to phenotype. This has been used to associate miR-137 pPGS with SZ risk(14) and neurological markers(15,16). Another recent implementation identified SZ and BP subjects with risk in pathways targetable by available pharmaceuticals, showing the utility of pPGS in targeted medicine(17). Among pathways relevant to psychosis, altered glutamate(18-22), GABA(21-23), dopamine(24-27), and serotonin(27-29) signaling have been strongly associated with psychotic disorders and symptoms.

There is a major opportunity to use pPGS to identify pathway contributions to psychosis symptoms. Here, we investigated the relationship of overall and neurotransmitter-associated PGS to psychotic disorder presentation at the diagnostic and endophenotypic level. We estimated overall and pathway-level PGS for a diverse cohort recruited from California following a first psychotic episode, and tested association between genetic burden, diagnosis, and clinical and neuroimaging endophenotypes. We found that overall and pathway PGSs were elevated in cases, pPGS was associated with specific endophenotypes, and unbiased clustering on phenotypes outperformed diagnosis in associations with pPGS and treatment response. Our results demonstrate the power of pPGS to link genetic and neurobiological underpinnings of psychotic disorders.

## Methods

### Study participants

Psychosis subjects were all outpatients within two years of their first psychotic episode. Subjects were selected from an ongoing psychosis research cohort, which includes 196 first-episode SZ-spectrum patients, 53 patients with first-episode BP with psychotic features, and 135 controls aged 12-38. After all quality control (QC), 119 SZ, 39 SA, 48 BP, and 115 controls were included for this study from an ongoing early psychosis research cohort. The study was approved by the University of California, Davis, Institutional Review Board and all subjects gave written consent and were paid for their participation.

### Psychiatric and neuroimaging phenotyping

All participants were assessed using the Structured Clinical Interview for the DSM-IV-TR (SCID I/P)(30). Clinical interviews were conducted by clinicians with masters or doctoral degrees trained to high reliability (kappa > .70; range = .70-1.0). All patients were followed longitudinally and diagnoses were confirmed 6 months after ascertainment. Exclusion criteria for all groups included: Wechsler Abbreviated Scale of Intelligence (WASI) IQ score below 70, alcohol or drug dependence or abuse within 3 months before testing, positive urine toxicology screen for illicit drugs, prior head trauma worse than a Grade I concussion, or contraindication to MRI scanning. Control subjects were excluded for the following additional criteria: any lifetime diagnosis of an Axis I or Axis II disorder or any first-degree relatives with a psychotic disorder. Before testing, a detailed description of the study was provided and written informed consent obtained.

Subjects were evaluated on the Global Assessment of Functioning (GAF)(31), Global Social Functioning scale (GSF)(32), Global Role Functioning scale (GRF)(33), Young Mania Rating Scale (YMRS)(34), Scale for the Assessment of Positive Symptoms (SAPS)(35), Scale for the Assessment of Negative Symptoms (SANS)(35), and Brief Psychiatric Rating Scale (BPRS)(36). Reality distortion, poverty symptoms, and disorganization scores were defined from the BPRS, SAPS, and SANS(37). Treatment response was defined as > 20% decrease in BPRS from baseline(38).

The GSF and GRF were measured at multiple time points; for each scale, we computed an average of the highest and lowest values measured for each subject during the past year and proceeded with these values for subsequent analyses. Finally, we removed item #8 (“Content”) from scores for the YMRS, as this question asks specifically about positive psychotic symptoms and can skew YMRS scores for subjects with schizophrenia. As such, scores on the YMRS used in these analyses more directly represent mania-specific symptoms. At baseline, all patients had BPRS scores >=5 to offer sufficient resolution to detect a 20% improvement in score at follow-up. For treatment response calculation, BPRS was rescaled to a lowest score of zero (i.e. score of 24=score of 0)(39).

Behavioral and neuroimaging methods are described previously(40). Consequently, we present these methods in a condensed form. The AX-Continuous Performance Task (AX-CPT)(41) was performed during fMRI. In short, the task requires participants to respond to a series of cue and probe letters and correctly identify the target pair (“AX” trials) while correctly rejecting other pairs. The frequency manipulation of trial types creates a prepotent tendency to make a target response when the “X” probe letter is presented. Consequently when a non-A cue is presented and followed by an X (i.e. “BX” trials) the participant must engage proactive control to both retain the goal, keep the incorrect cue in mind, and correctly reject the trial at the probe phase. Participants were excluded if performance did not meet a minimal threshold(42). The primary behavioral measure used for this study is d’-context, which represents a contrast of AX hits versus BX false alarms.

Functional Blood Oxygenation Level Dependent (BOLD) data were acquired using a 1.5T GE Signa and 3.0T Siemens TimTrio. Two regions of interest were defined *a priori* and comprised CueB versus CueA contrast, reflecting high versus low cognitive control-related activity. Specifically, bilateral dorsolateral prefrontal cortex (DLPFC) and bilateral superior parietal cortex (SPC) were defined as 5mm radius spheres based on coordinates from two independent datasets(43,44). All fMRI data were preprocessed using SPM8 (Wellcome Dept, of Imaging Neuroscience, London) and included slice timing correction, realignment, normalization to the Montreal Neurological Institute (MNI) template, and smoothing with an 8mm FWHM Gaussian kernel. Individual runs were excluded when framewise displacement measures of movement exceeded 0.45mm (calculated with https://fsl.fmrib.ox.ac.uk/fsl/fslwiki/FSLMotionOutliers) and whole subjects were excluded if more than half of their data exceeded this threshold. All trial types were modeled (CueA, CueB, AX, AY, BX, BY) and correct responses were used to create first-level images.

Based on the exclusion criteria mentioned above, five controls, nine patients with schizophrenia, and two patients with bipolar disorder were excluded due to excess motion. Two controls, five patients with schizophrenia, and two patients with bipolar disorder were excluded for poor behavioral performance. Finally, three controls and six patients with schizophrenia were excluded for other reasons, including scanning artifacts, scanner failure, or button pad failure.

### Genotyping, Quality Control, and Imputation

Subjects underwent blood draws using PAXgene blood DNA tubes, which were subsequently stored at −80 °C until DNA extraction. Tubes were thawed at 37 °C for 15 minutes before extracting DNA using the Qiagen QIAamp DNA Blood Mini Kit. The protocol was followed as written with minor modifications: volumes of blood, protease, lysis buffer, and ethanol were tripled prior to binding the DNA to the spin column; the final elution incubation was carried out at 50 °C for five minutes; and DNA was eluted in 100 μl of nuclease-free water. After DNA extraction, samples were cleaned on the Zymo Research Genomic DNA Clean & Concentrator-10 kit with minor modifications: an extra two-minute spin in a clean collection tube was added after completing the wash steps; samples were incubated at 50 °C for five minutes at the elution step; and DNA was eluted in 100 μl of nuclease-free water. After cleaning, all samples were analyzed on a spectrophotometer and verified to have concentrations ≥ 50 ng/μl and 260/280 and 260/230 ratios ≥ 1.70.

DNA was genotyped using the Illumina (San Diego, California) Infinium PsychArray-24 Kit at the UC Davis DNA Technologies Core. Initial QC was performed using Illumina GenomeStudio following published guidance(45,46). Additional quality control was applied using PLINK. First, variants with greater than 5% missingness were removed. We confirmed that no individuals had more than 1% of SNPs missing, nor did we observe any mismatch between the genetic sex inferred by PLINK and subjects’ self-reported sex. We removed variants which had significantly different missingness rates between cases and controls (p < 0.001) or variants which indicated significant deviation from Hardy-Weinberg equilibrium (p < 1e-6). No severe heterozygosity outliers were observed. A small number of pairs of individuals appeared to be related; in these cases one subject from each pair was randomly excluded from subsequent analyses. Following imputation, the QC filters outlined above were performed again. Additionally, variants with imputation quality INFO scores less than 0.7 or empirical INFO scores less than 0.8 were removed.

PLINK (v1.9) was used to calculate genetic principal components (PCs) in the 1000 Genomes(47) Phase 3 dataset, which were projected onto our sample. Samples were submitted to the Michigan Imputation Server for genotype imputation, using the full 1000 Genomes(47) Version 3 dataset as the reference panel. Following all QC, 7,608,150 SNPs were available across 338 unrelated subjects. Following additional review, 14 subjects with schizophreniform disorder and 2 subjects with schizotypal disorder that had initially been included were excluded from subsequent analyses due to insufficient numbers, such that the final number of subjects was 321 as described in “Study Participants.”

We additionally checked for presence of known copy number variants (CNVs) of high penetrance for schizophrenia risk in our samples using iPsychCNV5 and PennCNV(48-50), using default parameters. CNVs called by both software were retained as consensus calls for further analysis. This resulted in a total of 333 CNVs across all subjects. We cross-referenced these results with genome-wide significant CNV loci associated with SZ(51) and filtered for CNVs in our sample that overlapped with at least 50% of one of these previously identified SZ risk CNV loci. One subject with SZ appeared to have a previously described SZ risk CNV: 15q11.2 deletion. As the role of deletion at 15q11.2 is currently of uncertain significance in schizophrenia risk(52,53), we retained this individual for all analyses.

### Polygenic Score Calculation

**Figure 1:**
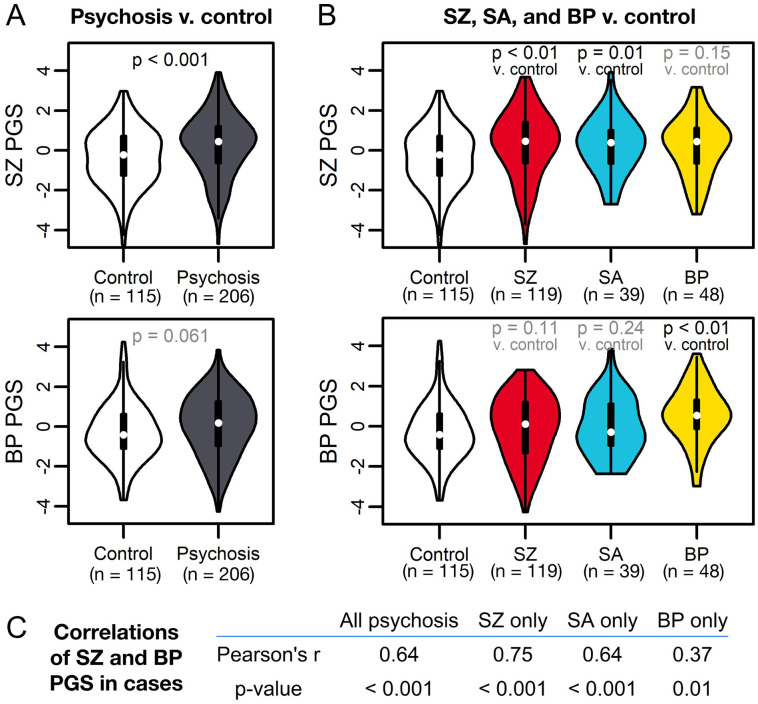
SZ and BP PGS are associated with psychotic disorder diagnoses. (A) SZ PGS (top) is significantly associated with psychosis case status, while BP PGS (bottom) does not reach significance. (B) SZ PGS (top) PGS is associated with SZ and SA status versus controls, but not BP status. Conversely, BP PGS (bottom) is associated with BP status versus controls, but not SZ or SA status. For each (A) and (B), the top row represents a comparison of SZ PGS across diagnostic groups, while the bottom row represents a comparison of BP PGS across diagnostic groups. (C) Pearson’s correlations of SZ and BP PGS in psychosis cases show moderate-to-high correlations between PGS for all psychosis subjects, as well as for people in individual diagnostic groups. This correlation is somewhat lower for BP subjects.

Using Psychiatric Genomics Consortium GWAS summary statistics for SZ (2021)(54) and BP (2022)(55), we employed PRS-CS(56) to calculate SZ and BP PGS, with the phi parameter set to 0.01 as recommended without a validation sample. PRS-CS applies shrinkage to optimize PGS prediction and may not be applicable when restricting PGS to genes from specific pathways. Therefore, we used the PRSet function from PRSice(57) to calculate pPGS for the four neurotransmitter pathways using SZ GWAS summary statistics(54), as our subjects were weighted toward SZ diagnosis and showed high correlation between SZ and BP PGS (Fig. 1). As recommended by the authors of PRSet, in order to avoid eliminating SNPs in certain genes, no p-value filter was applied when calculating pPGS. However, in order to choose an optimal R^2^ cutoff for clumping, we tested five potential values (0.1, 0.3, 0.5, 0.7, 0.9). In the subset of European ancestry samples, we found that using all SNPs as input, a clumping R^2^ of 0.7 maximized prediction of case-control status of the overall SZ PGS. Thus, this value was used when calculating pPGS for each of the four pathways. Otherwise, default parameters of PRSet were retained, with the 1000 Genomes(47) European subset used as the LD reference panel.

To reduce bias from population stratification, we employed an ancestry-specific standardization process to model PGS in a mixed-ancestry sample. First, we assigned each subject to their nearest 1000 Genomes(47) population by minimizing Euclidean distance to population centroid in 20-dimensional PC space, as recommended by Prive et al.(58) Then, we calculated overall PGSs and pPGSs for all samples in the 1000 Genomes(47) dataset using the same SNPs and weights used in our target sample. Finally, we z-score normalized PGS for each subject relative to their matching 1000 Genomes(47) ancestry. Thus, PGSs are standardized within ancestry-matched population such that a subject with CEU ancestry and a standardized PGS of 1 has a raw PGS one standard deviation above the average of the 1000 Genomes(47) CEU samples.

### pPGS pathways

Pathways include genes relevant in glutamate, GABA, dopamine, and serotonin. Genes were sourced from KEGG(59), REACTOME(60), and AmiGO(61) by searching for pathways and ontologies that include these neurotransmitters or variations on them (e.g. “glutamatergic”). The complete gene list is in Table S1.

### Unbiased clustering

Phenotypes were z-score normalized within cases. Subjects were clustered on all phenotypes. We used all subjects with complete phenotype data (n = 90) to calculate the optimal number of clusters using the NbClust R package(62). Estimates converged on three clusters. Subsequently, we used all subjects with greater than 50% of phenotype variables available (n = 167) for k-means clustering using the flipCluster R package(63).

### Statistical analysis

All analyses were completed in R(64). One BP subject was excluded as an outlier from analyses based on Cook’s distance near 1 in regression models. Correlation analyses and plots were produced using the psych(65) and corrplot(66) packages. Violin plots were produced using the vioplot package(67).

Nagelkerke’s R^2^ was calculated for variance explained in logistic regression models of disease status by PGS using the RMS R package(68). All phenotypes were tested against PGS and the following covariates in regression models: chromosomal sex, age, self-reported race, self-reported ethnicity, and the first four genetic PCs. Protocol/scanner type was controlled for in cognitive control analyses. Overall SZ PGS was included as a covariate for endophenotype analyses (Fig. 3). Models of continuous variable phenotypes against PGS are represented with partial regression plots.

For cluster versus diagnosis model comparison (Fig. 4E), we regressed PGSs separately against clusters, plus standard covariates, and against diagnoses, plus covariates. In separate analyses (Fig. 4F), we regressed PGS against both cluster and diagnosis in the same model, as well as covariates. BP and Cluster 3 were the reference levels for, respectively, diagnosis and cluster.

### Multiple testing corrections

**Figure 2:**
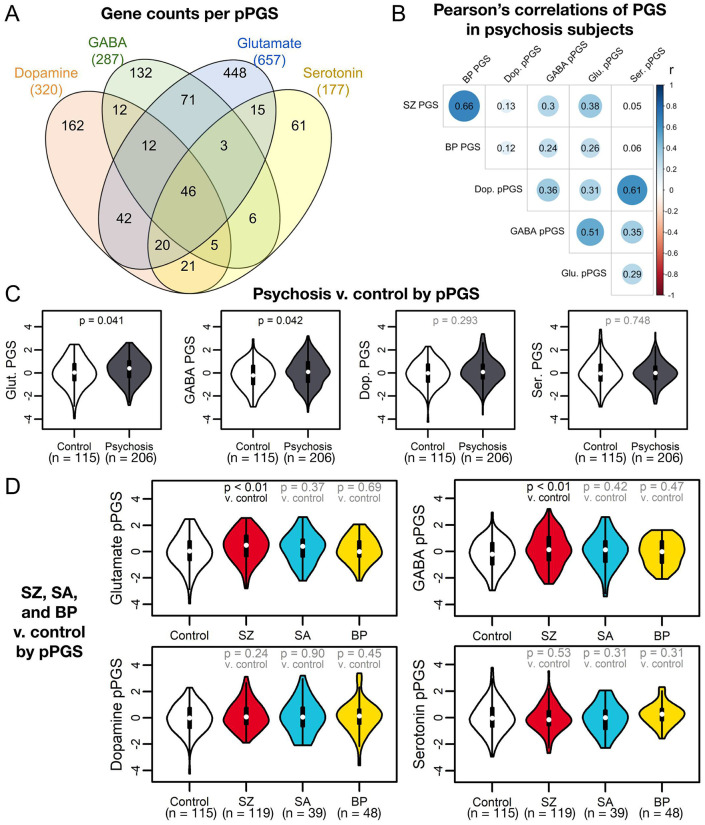
Pathway polygenic scores (pPGS) based on four main neurotransmitter systems. (A) KEGG(59), AmiGO(61), and REACTOME(60) were searched for genes associated with glutamatergic, GABAergic, dopaminergic, and serotonergic neurotransmission. (B) pPGS developed from these gene sets showed substantial correlation in psychosis subjects when regressing against the first four genetic principal components. All correlations were significant with p-value < 0.05 except for the correlations between serotonin pPGS and the overall SZ and BP PGS. (C) Glutamate and GABA pPGS were able to separate psychosis cases from controls and passed FDR < 0.10. (D) Glutamate and GABA pPGS much more strongly separated SZ cases from controls (FDR < 0.10) than either SA or BP subjects from controls.

**Figure 3:**
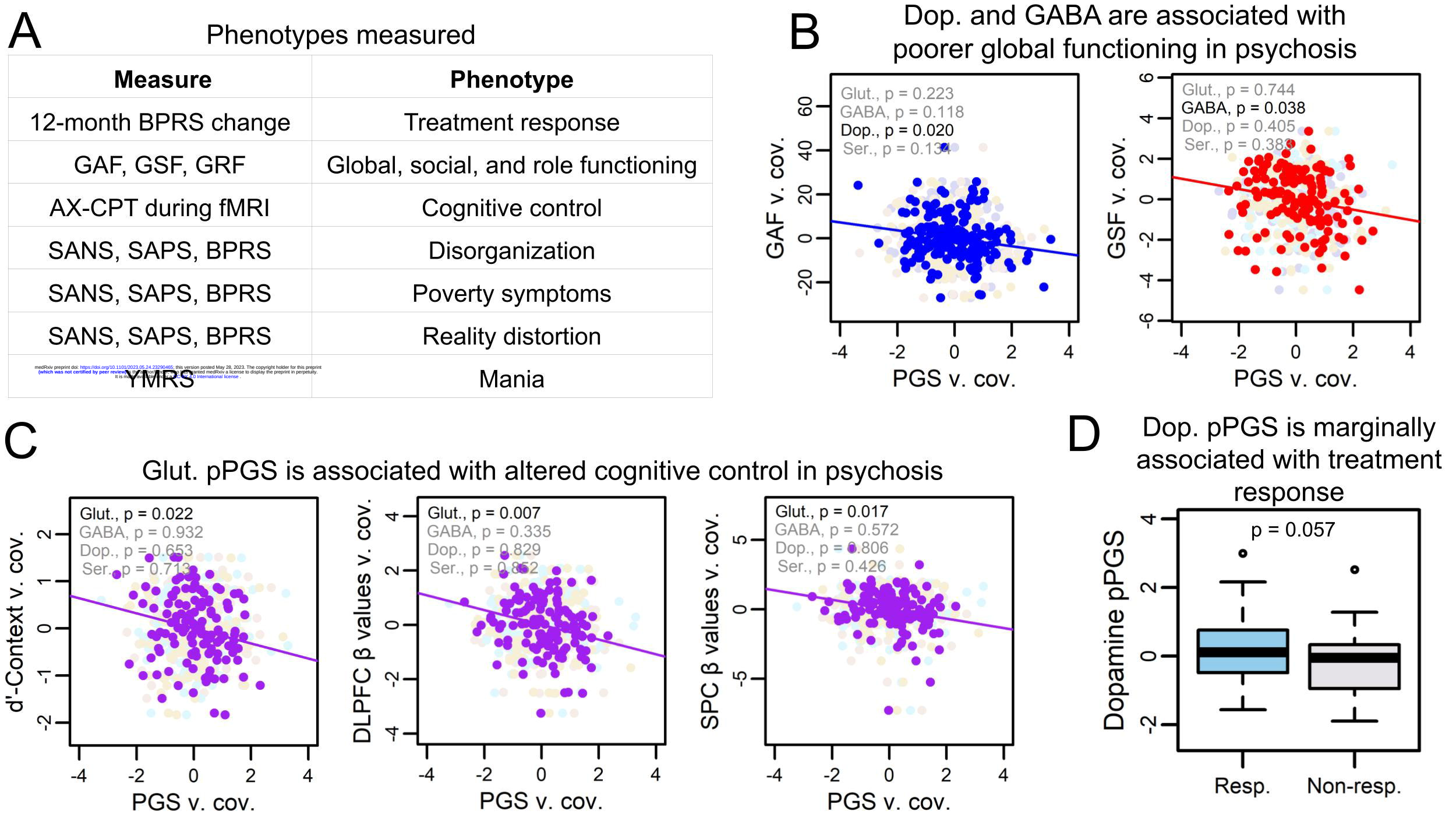
pPGSs identify mechanisms for endophenotypes of psychotic disorders. (A) Subjects were phenotyped on a range of psychological, clinical, and neurological measures. (B) Dopamine and GABA pPGS were associated with poorer global functioning in psychosis subjects when controlling for overall SZ PGS. Only the association with between dopamine and global functioning passed FDR < 0.15. (C) Glutamate pPGS showed strong and significant associations with poorer performance and reduced cortical activation in psychosis subjects during the AX-CPT cognitive control task when controlling for overall SZ PGS. These relationships passed FDR < 0.15. (D) Treatment response showed a slight association with increased dopamine pPGS, though this did not pass FDR < 0.15. (Resp. = responder to treatment, non-resp. = non-responder to treatment)

Multiple testing corrections were applied for analyses of phenotype, including diagnosis and case status, by pathway PGS (Figs. 2, 3). Given the correlated structure of phenotypes and of pPGS in our subjects, we used the poolr R package(69) to calculate the effective number of statistical tests following Galwey (2009)(70). This methodology identified effectively three (of initially four) independent pPGS variables and eight (of initially ten) independent endophenotype variables. As differential diagnoses of SZ, SA, or BP were mutually exclusive, they were not adjusted for correlated structure. Total tested dependent variables were multiplied by total tested independent variables such that findings reported in Figure 2 were corrected for either three (case status by three pPGS) or nine (three diagnoses by three pPGS) total tests, and findings reported in Figure 3 were corrected for 24 total tests (eight endophenotypes by three pPGS) using the Benjamini-Hochberg(71) false discovery rates reported in Results..

### Permuted null pathway calculation (Fig. S2)

For each of the pathways in our main analyses, we constructed 10,000 matched null gene sets with an equivalent number of genes, chosen such that the probability of a gene’s inclusion is proportional to its length. We then calculated pPGS for these 40,000 permuted null gene sets in the same way as described above, i.e. using PRSet to calculate pPGS for our target sample and the 1000 genomes sample with weights from the most recent SZ GWAS and standardizing the target sample pPGS within each ancestry relative to the matched-ancestry 1000 genomes samples.

## Results

### Demographics

Subjects were demographically heterogenous and well matched across cases and controls (Table 1).

### Overall and pathway PGSs predict diagnostic status

We first tested whether overall PGS was associated with psychosis status. SZ PGS was associated with case status (OR = 1.37 [CI: 1.15,1.63]; p = 3.7x10^−4^) (Fig. 1A) and explained 5.2% of variance in status (p = 4.5x10^−4^), similar to previous reports(72-76) ( Fig. S1). This association was stronger in SZ (OR = 1.50 [CI: 1.22,1.84]; p = 1.3x10^−4^) and SA (OR = 1.59 [CI: 1.11,2.26]; p = 0.01) (Fig. 1B). While BP PGS was not significant in cases overall, it was elevated in BP subjects (OR = 1.47 [CI: 1.11,1.93]; p < 0.01) (Figs.1A,B). SZ and BP PGS were moderately correlated in cases (Fig. 1C). Thus, overall PGS captured both transdiagnostic and diagnosis-specific genetic risk for psychotic disorders, consistent with a strong literature on partial genetic overlap between SZ, SA, and BP(6,7,77-79).

### Association of pPGS with phenotypes

To dissect genetic contributions of biological pathways to clinical and neurobiological phenotypes, we calculated pPGS for four neurotransmitter systems relevant to psychosis: glutamate(18-22), GABA(21-23), dopamine(24-27), and serotonin(27-29) (Fig. 2A, Table S1). These showed moderate intercorrelations and correlations with overall PGS (Fig. 2B).

We first tested association with psychosis status. Glutamate and GABA pPGS explained a substantial portion of variance in disease status (R^2^ = 0.02-0.04, p < 0.05) ( Fig. S2). Glutamate and GABA pPGS were significantly elevated in cases (both: OR = 1.3 [CI: 1.1-1.6]; p = 0.04) (Fig. 2C), specifically SZ (both: OR = 1.4 [CI: 1.1-1.8]; p = 0.01) (Fig. 2D); these findings passed multiple testing corrections at a false discovery rate (FDR) < 0.10. This implies disease status in our cohort is partially explained by glutamatergic and GABAergic genetic risk.

### pPGSs are associated with endophenotypes in cases

We next tested if PGS was associated with symptom variation across 10 phenotypes within subjects with a psychotic disorder (Fig. 3A). Overall PGSs were not significantly associated with measured phenotypes ( Fig. S2). However, we found associations of pathway-specific risk to six phenotypes in social functioning, global functioning, cognitive control, and treatment response. While no endophenotype associations passed multiple testing corrections at FDR < 0.10, all but social functioning and treatment response passed FDR < 0.15.

Dopamine and GABA pPGS were associated with, respectively, poorer global (β = −1.18 [CI: −3.32,−0.29]; p = 0.02) and social (β = −0.26 [CI: −0.05,−0.01]; p = 0.04) functioning (Fig. 3B), though the social functioning association did not pass FDR < 0.15. Perhaps the most robust finding was a strong association between glutamate pPGS and cognitive control in cases (β = −0.16 [CI: −0.30,−0.02] to −0.34 [CI: −0.62,−0.06]; p = 0.01-0.02) (Fig. 3C). This implies that perturbations in glutamate may be a determinant of poor cognitive control in psychosis subjects, recapitulating a robust literature associating glutamate with cognitive control(80-83).

Of 63 subjects with available treatment response data, 35 were responders, consistent with reported efficacy rates(84). We hypothesized subjects with higher dopaminergic risk would be more likely to respond to treatment, as they might be enriched for the kinds of biological differences that dopamine-targeting antipsychotics can address. While power to identify an association was reduced due to subsetting of our cohort, treatment response showed an association with higher dopamine pPGS (OR = 1.88 [CI: 0.98,3.59]; p = 0.057) (Fig. 3D), though this did not pass FDR < 0.15. This adds onto a strong literature associating dopamine with antipsychotic efficacy, providing evidence that an accumulation of dopaminergic risk may be associated with treatment efficacy.

### Unbiased phenotype clustering of psychosis cases and genetic burden

Biotype-level grouping of psychosis cases, such as by B-SNIP(9), may advance the use of biological data to better model psychopathology. We hypothesized a biotype-approach might yield stronger relationships with PGS compared to diagnosis. Toward this goal, we used k-means clustering to group endophenotype presentation among cases. We clustered subjects on z-score normalized phenotype data into three groups following cluster optimization using the NbClust R package(62).

**Figure 4:**
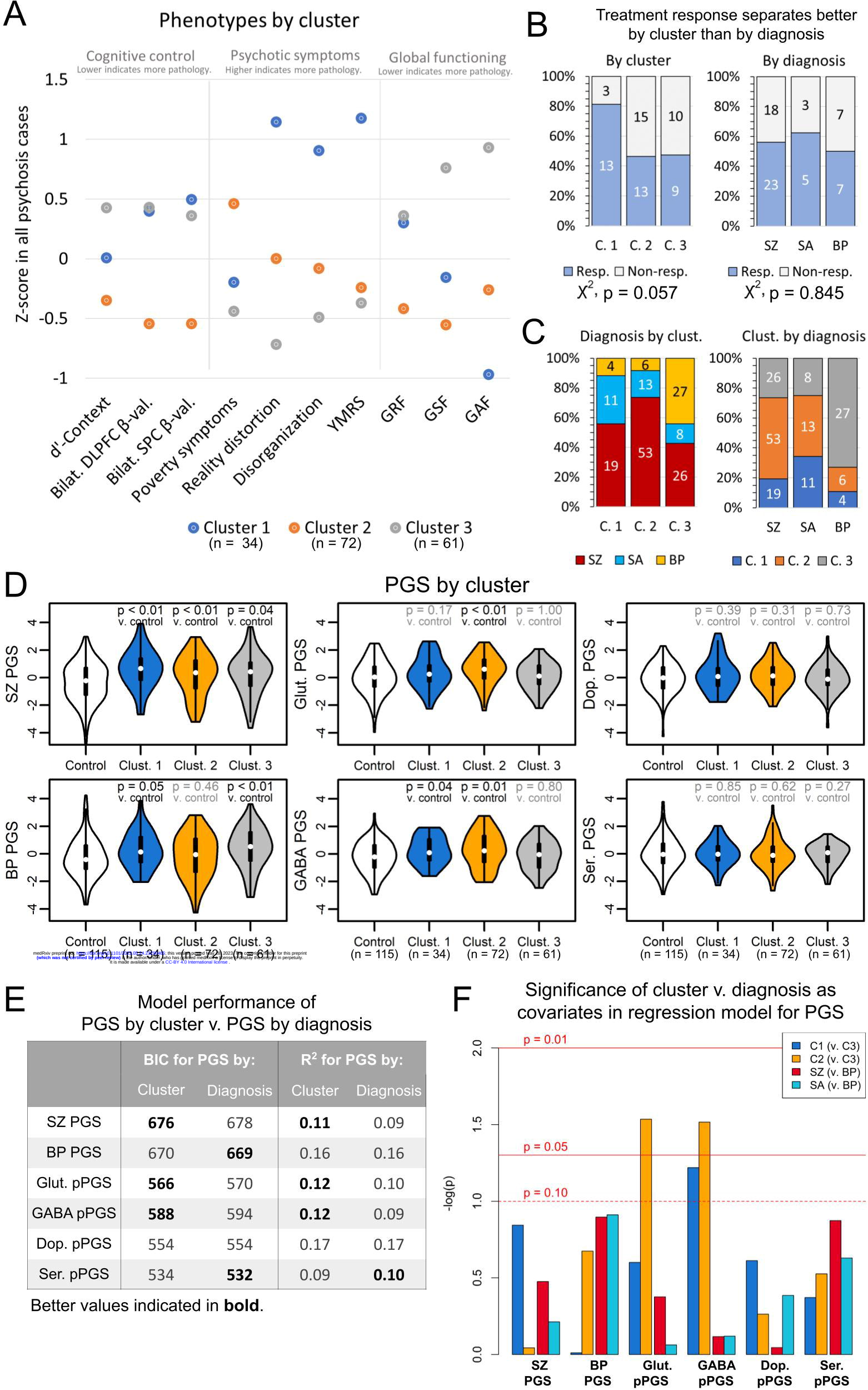
Psychosis subjects separate into clusters based on symptom profiles. (A) Psychosis subjects separate into three clusters that are characterized by (Cluster 1) mania, disorganization, reality distortion, and moderate impairments in cognitive control; (Cluster 2) high poverty symptoms, poor social functioning, and strong deficits in cognitive control; and (Cluster 3) overall low levels of pathology. (B) Cluster 1 showed enrichment for treatment responders. In contrast, treatment efficacy did not separate by diagnosis. (C) Diagnoses are mixed across clusters. (D) SZ and GABA PGS are elevated in Clusters 1 and 2. BP PGS is elevated in Clusters 1 and 3. Glutamate pPGS is elevated in Cluster 2. (E) Models regressing PGS against cluster perform better for glutamate and GABA PGS than models regressing PGS against diagnosis. Models using cluster also perform better in predicting SZ PGS, though models using diagnosis better predict BP and serotonin PGS. (F) Glutamate and GABA PGS are significantly associated with cluster membership when controlling for diagnosis. (C. 1 = Cluster 1, C. 2 = Cluster 2, C. 3 = Cluster 3, resp. = responder to treatment, non-resp. = non-responder to treatment)

Clusters showed distinct symptom profiles (Fig. 4A, Table S2). Cluster 1 was distinguished by high mania, disorganization, and reality distortion, and moderate impairments in cognitive control. Cluster 2 had deficits in negative symptoms, showed poor outcomes in role and social functioning, and had the poorest measures of cognitive control. Cluster 3 had low pathology. Consistent with its high degree of positive symptoms, Cluster 1 showed enrichment for treatment responders relative to Clusters 2 and 3, though this did not reach significance (p = 0.057) (Fig. 4B). In contrast, treatment response showed no separation by diagnosis (p = 0.845). Clusters were demographically and diagnostically heterogenous (Fig. 4C, Table S2), though Cluster 3 captured most BP subjects. This may indicate our clusters better distinguished transdiagnostic variation in SZ and SA subjects, though the presence of BP subjects in Clusters 1 and 2 suggests there is merit to psychosis-spectrum endophenotypic stratification for these subjects as well.

We next tested associations between cluster and PGS (Fig. 4D, Table S2). SZ PGS was associated with all clusters (OR = 1.28 [CI: 1.01-1.62] to 1.94 [CI: 1.31-2.89]; p = 0.001-0.039). BP PGS was strongly associated with Cluster 3 (OR = 1.42 [CI: 1.11-1.81]; p = 0.006), consistent with its preponderance of BP subjects, though also with Cluster 1 (OR = 1.40 [CI: 1.01-1.96]; p = 0.046). Cluster 3 was not associated with any pPGS, suggesting genetic risk may lie outside of neurotransmitter-linked loci. In contrast, GABA pPGS was elevated in Clusters 1 and 2 (OR = 1.47 [CI: 1.09-1.98] to 1.59 [CI: 1.02-2.49]; p = 0.011-0.040) while glutamate pPGS was only elevated in Cluster 2 (OR = 1.71 [CI: 1.24-2.37]; p = 0.001). This may have implications for the symptom profiles of these clusters. Cluster 2 showed strong negative symptoms, which are thought to largely arise from glutamatergic dysfunction(85,86). Cluster 2 also showed deficits in social and role functioning, which are predicted by negative symptoms(87), and strong cognitive control deficits. pPGS findings suggest Cluster 2’s symptoms may be more specific to GABAergic and glutamatergic risk, consistent with the hypothesis of an imbalance between these neurotransmitters in psychosis(22).

We next compared explanatory power of our clusters versus diagnoses to model genetic risk in our cohort. For this, we defined models regressing PGSs against either cluster or diagnosis. Per Bayesian information criteria and R^2^ (Fig. 4E), models using clusters performed better than models using diagnosis to predict glutamatergic, GABAergic, and overall SZ PGS. In contrast, overall BP PGS and serotonin pPGS were somewhat better modeled by diagnosis, and there was no difference for dopamine pPGS. We next regressed PGS against both cluster and diagnosis as covariates to test independence between diagnosis and cluster (Fig. 4F). Again, glutamate and GABA pPGS were significantly associated with Cluster 2 when including diagnosis in the model (β = 0.46 [CI: 0.05,0.88] to 0.49 [CI: 0.05,0.94]; both p = 0.03). Neither cluster nor diagnosis was independently associated with other PGSs. These results provide evidence for the biological validity of these clusters overall and within the context of molecular pathways. In particular, glutamate and GABA pPGS seem to be more closely associated with cluster than with diagnosis.

## Discussion

We tested how overall and pathway-specific PGS are related to diagnosis and variation in psychosis symptoms in a diverse patient sample. We curated gene sets representing four major neurotransmitter systems(59-61) and calculated pPGS for SZ variants in these pathways. We also calculated overall SZ and BP PGS. Overall PGS, as well as glutamate and GABA pPGS, were associated with case status. pPGSs outperformed overall PGS in explaining endophenotypes: dopamine was associated with treatment response and global functioning, GABA was associated with social functioning, and glutamate was associated with cognitive control. Unsupervised clustering identified three phenotypically distinct groups of psychosis subjects that differed predominantly on positive symptoms, negative symptoms, cognitive control, and global functioning. Though our data largely represent different phenotypic information than that used by B-SNIP, we reproduced those findings which our measures can capture and generated new insights on the relationship between biotypes and genetic burden. Like B-SNIP, we identified three diagnostically mixed endophenotypes that separate on general impairment and cognitive control. Stratification was supported by distinct PGS profiles. Notably, Cluster 2, which showed primary deficits in cognitive control, negative symptoms, and social functioning, had elevated glutamatergic and GABAergic risk. Treatment efficacy separated by cluster and model comparisons showed that GABA and glutamate pPGS were better modeled by cluster than diagnosis. Our results link overall and pathway-specific partitions of genomic risk to transdiagnostic endophenotypes of psychotic disorders.

Our study features limitations. Notably, our cohort is smaller than many genomics studies, though our deep level of phenotyping is a strength and generally not possible for the larger or combined cohorts needed for GWAS. Focusing on PGS rather than SNP discovery mitigates these limitations, though limits our study to integration of previously-defined associations. We tested *a priori*-defined and hypothesis-driven pathways in the present study, however, there are certain to be other contributing pathways not investigated here. Our demographic heterogeneity, while a strength, presents methodological challenges. Thus, we controlled for race, ancestry, and genetic PCs in analyses. We further z-score normalized PGSs to subjects’ genetically matched(58) 1000 Genomes(47) ancestries. Finally, k-means clustering will identify clusters even when the true nature of the data is continuous or cluster differences are not biologically relevant. The identification of unique PGS profiles for our clusters, as well as by-cluster differences in treatment response, provide auxiliary evidence supporting separate mechanisms driving distinct clusters of primary symptoms. Comparisons of clusters versus diagnoses show that clusters better predict glutamate and GABA pPGS in our cohort. Additionally, we replicate key findings from previous clustering attempts on psychosis subjects(9).

Our results show the utility of pPGS to interrogate convergent neurobiological mechanisms underlying disease phenotypes. The neurotransmitter systems tested here have been robustly associated with psychotic disorders(18-29). However, their relationship to specific symptoms have not been elucidated. Mechanisms underlying cognitive and negative symptoms in psychosis are particularly poorly understood, despite being some of the strongest predictors of poor outcomes in psychotic disorders(88-91). Our results robustly support a role for glutamate dysfunction in cognitive, as well as negative and social, symptoms in psychosis. This provides evidence that the biological basis of these symptoms may be partly explained by genetic risk driving glutamatergic abnormalities, which has been widely hypothesized. Our study and findings represent a model for going beyond overall genetic burden in investigation of the genetic components of psychosis, and, in particular, of maximizing power in smaller but deeply characterized cohorts. While our findings will need to be replicated, they offer a promising look into how genetic burden can be partitioned and can explain specific psychosis diagnosis and endophenotypes. Our work is a step towards a more precise understanding of convergent molecular mechanisms underlying psychotic symptoms, which is critical for realizing the promise of stratified medicine and targeted treatments for those suffering from psychotic disorders.

## Supplementary Material

Supplement 1

Supplement 2

## Figures and Tables

**Table 1:** Demographics of study subjects. Study subjects were racially and ethnically diverse across both cases and controls. Potential differences in demographics were considered when controlling for covariates in subsequent analyses. (s.d. = standard deviation)

	Cases (N)	(%)	Controls (N)	(%)
**Total**	206	64%	115	36%
**Diagnosis**				
Schizophrenia	119	58%	-	-
Bipolar Disorder	48	23%	-	-
Schizoaffective Disorder	39	19%	-	-
**Race**				
African American/Black	28	14%	7	6%
American Indian/Alaskan Native	2	1%	0	0%
Asian	15	7%	27	23%
Caucasian/White	131	64%	61	53%
Native Hawaiian/Pacific Islander	3	1%	1	1%
Multiple	23	11%	17	15%
Unknown	4	2%	2	2%
**Ethnicity**				
Hispanic/Latino	50	24%	17	15%
Non-Hispanic/Latino	151	73%	97	84%
Unknown	5	2%	1	1%
**Inferred 1000 Genomes superpopulation**				
African (AFR)	58	28%	19	17%
American (AMR)	20	10%	8	7%
East Asian (EAS)	14	7%	26	23%
European (EUR)	111	54%	53	46%
South Asian (SAS)	3	1%	9	8%
**Sex**				
Male	158	77%	67	58%
Female	48	23%	48	42%
**Mean age (s.d.)**	19.7 (4.1)		19.7 (4.2)	
