# Supplementary material for: Association of neurotransmitter pathway polygenic risk with specific symptom profiles in psychosis
Source: medRxiv. 2023 May 28:2023.05.24.23290465. Preprint. [Version 1] doi: 10.1101/2023.05.24.23290465 (PMC10246134; doi:10.1101/2023.05.24.23290465)
Supplement: Supplementary file 1 [file media-1.pdf]

Supplementary figures and tables

Fig. S1: Explanatory power of genome-wide PGS for disease status in our cohort is comparable to that previously reported in the literature.

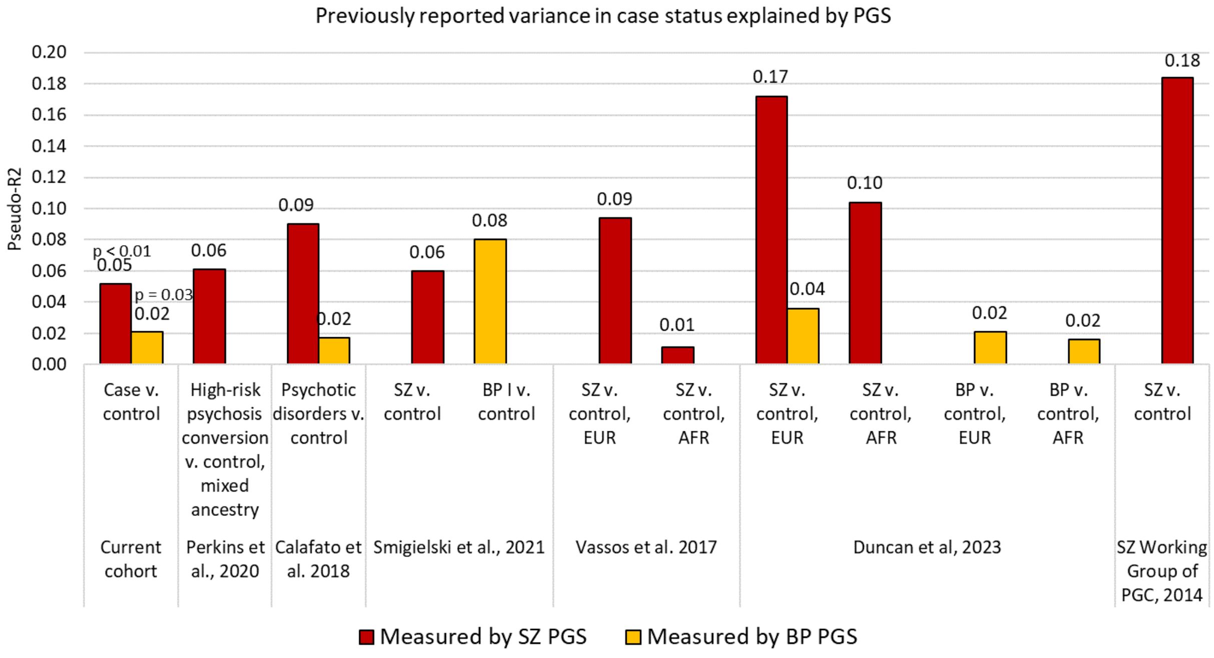

Fig. S2: Glutamate and GABA pPGSs explain partial variation in (A) overall psychosis status and (B) schizophrenia vs. control status. Red bar indicates Nagelkerke's R^2 for the neurotransmitter pPGS. pPGSs are plotted against a null distribution of 10,000 size-matched random gene sets.

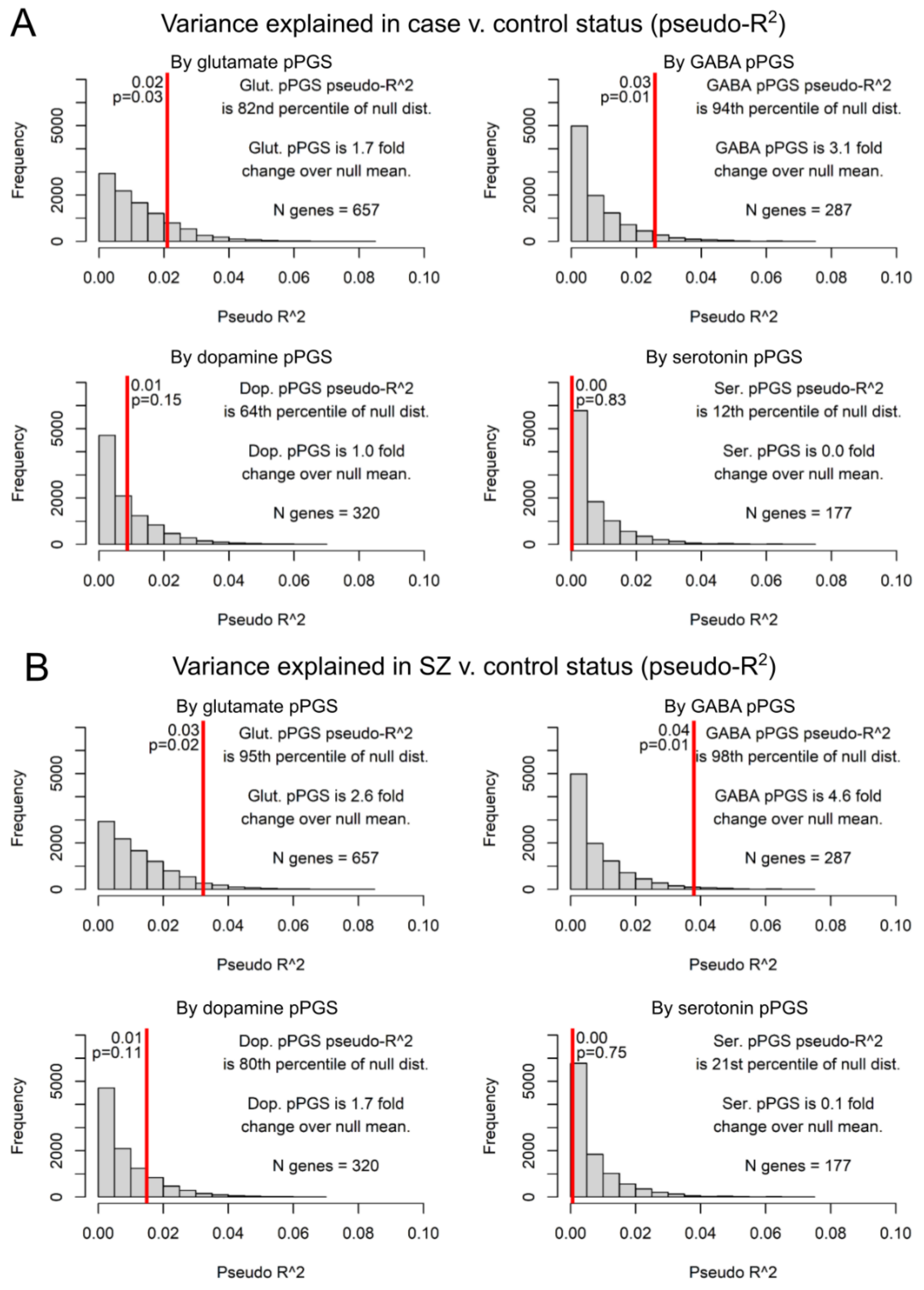

Fig. S3: Phenotypes measured in this study were not significantly associated with overall SZ or BP PGS in our cohort.

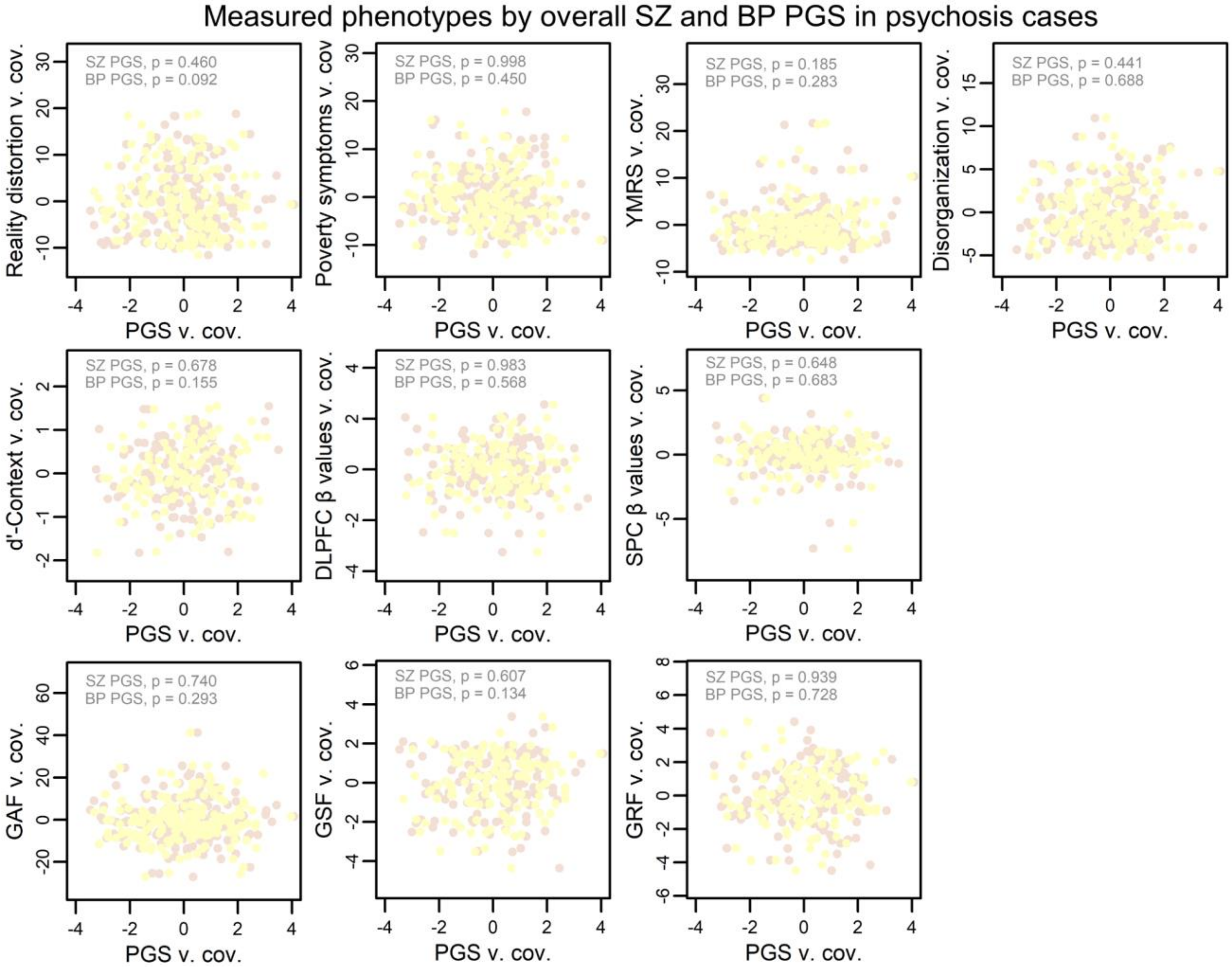

Table S1: Genes in neurotransmitter pathways
Data is available as a downloadable Excel file.

Table S2: Summary of k-means clusters. Clusters were demographically heterogeneous and showed distinct symptom and pPGS profiles.

	Cluster 1	Cluster 2	Cluster 3
**N**	34	72	61
**Diagnoses**	SZ: 19 (56%)<br>SA: 11 (32%)<br>BP: 4 (12%)	SZ: 53 (74%)<br>SA: 13 (18%)<br>BP: 6 (8%)	SZ: 26 (43%)<br>SA: 8 (13%)<br>BP: 27 (44%)
**Primary symptoms**	‣ Cog. control (moderate)<br>‣ Reality distortion<br>‣ Disorganization<br>‣ Mania<br>‣ Social functioning<br>‣ Global functioning	‣ Cog. control (severe)<br>‣ Poverty symptoms<br>‣ Role functioning<br>‣ Social functioning<br>‣ Global functioning	(mild deficits)
**Highest PGS**	‣ SZ PGS	‣ SZ PGS<br>‣ Glutamate PGS	‣ BP PGS
**Race**			
African American/Black	3 (9%)	13 (18%)	6 (10%)
American Indian/Alaskan Native	1 (3%)	0 (0%)	1 (2%)
Asian	2 (6%)	8 (11%)	3 (5%)
Caucasian/White	24 (71%)	42 (58%)	41 (67%)
Native Hawaiian/Pacific Islander	0 (0%)	2 (3%)	1 (2%)
Multiple/unknown	4 (12%)	7 (10%)	9 (15%)
**Ethnicity**			
Hispanic/Latino	10 (29%)	16 (22%)	46 (75%)
Non-Hispanic/Latino	24 (71%)	55 (76%)	14 (23%)
Unknown	0 (0%)	1 (1%)	1 (2%)
**Inferred 1000 Genomes SuperPopulation**			
AFR	8 (24%)	25 (35%)	12 (20%)
AMR	5 (15%)	7 (10%)	5 (8%)
EAS	1 (3%)	6 (8%)	5 (8%)
EUR	20 (59%)	32 (44%)	39 (64%)
SAS	0 (0%)	2 (3%)	.0 (0%)
**Sex**			
Male	28 (82%)	59 (82%)	45 (74%)
Female	6 (18%)	13 (18%)	16 (26%)
**Mean age (s.d.)**	20.4 (4.3)	19.1 (4.3)	20.0 (4.0)
